# Monthly Summary

**Published:** 1882-07

**Authors:** 


### MONTHLY SUMMARY.

*The Medicinal Value of Vegetables.*—A celebrated cook-book discusses the medicinal value of vegetables, as follows:

"Asparagus is a strong diuretic, and forms part of the cure for rheumatic patients at such health resorts as Aix-les-Bains. Sorrel is cooling, and forms the staple of that *soupe aux herbes* which a French lady will order for herself after a long and tiring journey. Carrots. as containing a quantity of sugar, are avoided by some people, while others complain of them as indigestible. With regard to the latter accusation, it may be remarked, in passing, that it is the yellow core of the carrot that it is difficult of digestion—the outer, a red layer, is tender enough. In Savoy, the peasants have recourse to an infusion of carrots as a specific for jaundice.

"The large, sweet onion is very rich in those alkaline elements which counteract the poison of rheumatic gout. If slowly stewed in weak broth, and eaten with a little Nepaul pepper, it will be found to be an admirable article of diet for patients of studious and sedentary habits. The stalks of cauliflower have the same sort of value, only too often the stalk of a cauliflower is so ill-boiled and unpalatable that few persons would thank you for proposing to them to make part of their meal consist of so uninviting an article. Turnips, in the same way, are often thought to be indigestible, and better suited for cows and sheep than for delicate people ; but here the fault lies with the cook quite as much as with the root. The cook boils the turnip badly, and then pours some butter over it, and the eater of such a dish is sure to be the worse for it. Try a better way. What shall be said about our lettuce? The plant has a slight narcotic action, of which a French old woman, like a French doctor, well knows the value, and when properly cooked it is really very easy of digestion."—*Med. Record.*

---

*Is This a New Dental Disease?*—A child, aged ten, whose teeth six months ago appeared to be all perfectly sound, came to me with tooth ache in the right lower canine. I found that a large portion of the enamel had disappeared from the front surface of the tooth, as if it had been chipped violently off; the dentine was exposed, but there was no softening or appearance of decay. The disease, which was commenced in several of the other incisor teeth, appears first as a small white spot in about the thickest part of the front surface of the enamel, which it seems to penetrate; find then, suddently disintegrating, this comes away, and exposes the remaining sensitive enamel and the dentine. This disease is altogether a different thing from the gradual decay or wear at the neck of the teeth, frequently met with in adults, for in this case the patient is only ten ; and, as far as I have been able to ascertain, the incisors and canines never have been known to decay in the manner above described. We are often at our wits' end to cope with that increasing prevalence of caries in the teeth of the very young; and if this be (as I fear it is) a new form of destructive energy, the sooner it is recognized the better.—*British Medical Journal.*

---

*Removal of Foreign Bodies from the Ear.*—In the Detroit *Lancet.* Dr. A. F. Kinne, after pointing out the defects in all the existing instruments and methods for the removal of foreign bodies from the ear, describes a simple instrument, devised by himself, for this purpose, which has answered admirably. It really consists of a minute, sharp-pointed, tenaculum-shaped hook, for the removal of foreign bodies from the nose. In order to remove a foreign body that has been pushed through the tympanum into the deeper recesses of the ear, it may, in some cases, become necessary to detach the auricle before an instrument can be brought to bear upon it. As a justification for this operation, he relates the following case :—

About twenty five years ago, I was called to a well grown boy whose ear had been torn off, close to his head, by a wagon wheel. The wound was ragged and dirty, and the bystanders thought the ear "no good;" but the skin in front of it was not tore through, and I decided to attempt its restoration, and succeeded. I pinned it on with cambric needles, annealed and curved. And, when I passed my ligature around the ends of these, I sought to bring the torn edges into close apposition, without drawing it tightly enough to interrupt the local circulation.

Of course, very complete access to the middle ear is gained by detachment of the auricle. And, as to the feasibility of the operation, two questions, and the only ones of any consequence, were settled well enough by the results of this incident. First, an ear will grow on again, even if the incisions by which it is detached are not smooth and clean; and second, the resultant scar is one of no consequence, especially when it is located behind the ear.—*Med. and Surg. Reporter.*

---

*Congenital Teeth.*—Dr. F. B. Harrington reports the following case in the Boston *Medical and Surgical Journal:*—

Two small white spots were accidently noticed on the lower jaw of a child, then twelve days old. The spots were supposed to be thrush. Four days later the mother complained that the child caused her great pain in the nipples while nursing. An examination of the mother s breasts did not show adequate cause. On looking into the child's mouth the two spots were found to be two sharp-edged incisor teeth. They were removed and appeared to be the right central and right lateral lower incisors.

